# Reduced Doses of Diatomaceous Earth and Basil Essential Oil on Stored Grain Against the Wheat-Damaging *Sitophilus oryzae*: Influence on Bread Quality and Sensory Profile

**DOI:** 10.3390/foods14040572

**Published:** 2025-02-09

**Authors:** Alessandro Bianchi, Priscilla Farina, Francesca Venturi, Francesca Trusendi, Guido Flamini, Roberta Ascrizzi, Sabrina Sarrocco, Sania Ortega-Andrade, Maria Cristina Echeverria, Barbara Conti, Isabella Taglieri

**Affiliations:** 1 Department of Agriculture, Food and Environment, University of Pisa, Via del Borghetto 80, 56124 Pisa, Italy; alessandro.bianchi@phd.unipi.it (A.B.); priscilla.farina@agr.unipi.it (P.F.); f.trusendi@studenti.unipi.it (F.T.); sabrina.sarrocco@unipi.it (S.S.); isabella.taglieri@unipi.it (I.T.); 2 Research Center Nutraceuticals and Food for Health (Nutrafood), University of Pisa, Via del Borghetto 80, 56124 Pisa, Italy; guido.flamini@unipi.it (G.F.); roberta.ascrizzi@unipi.it (R.A.); 3 Department of Pharmacy, University of Pisa, Via Bonanno Pisano 12, 56126 Pisa, Italy; 4 Centre for Climatic Change Impact, University of Pisa, Via del Borghetto 80, 56124 Pisa, Italy; 5 Department of Biotechnology, Universidad Técnica del Norte, Avenida 17 de Julio 5–21 y General José María Córdova, Ibarra 100150, Ecuador; smortega@utn.edu.ec (S.O.-A.); mecheverria@utn.edu.ec (M.C.E.)

**Keywords:** Dryophtoridae, *Ocimum basilicum*, stored product pests, wheat, bread, sensory analysis, volatile organic compounds

## Abstract

Stored grain pests like *Sitophilus oryzae* pose significant challenges to food security and quality, necessitating eco-friendly pest management strategies. This study investigates the combined efficacy of reduced doses of diatomaceous earth (DE) and basil (*Ocimum basilicum* L.) essential oil (EO) as an alternative to conventional pesticides. Laboratory trials evaluated the effectiveness of the treatments—DE, EO, and a mixture of both (at halved doses)—against *S. oryzae* in wheat, alongside their impact on bread quality and sensory attributes. Results showed that DE and the DE + EO at halved doses combination achieved over 82% pest mortality, comparable to standard DE doses but with reduced mechanical and environmental drawbacks. EO alone demonstrated limited insecticidal activity. Bread made from treated wheat retained high sensory acceptability, with DE enhancing elasticity and crumb aroma. EO-enriched bread exhibited a complex aromatic profile due to methyl chavicol, though with reduced crumb elasticity and a slightly bitter aftertaste. Shelf-life assessments indicated that DE and DE + EO at halved doses extended mold-free storage by one day compared to untreated bread. These findings highlight the potential of combining DE and EO at reduced doses to manage stored grain pests sustainably, aligning with integrated pest management (IPM) and organic farming principles, while preserving the technological and sensory qualities of derived food products.

## 1. Introduction

Wheat and its bakery derivatives, rich in calories, minerals, vitamins, dietary fibers, beneficial bioactive compounds, and essential amino acids, are a crucial part of human staple nutrition [1,2,3]. Among these, bread plays a pivotal role, although its nutritional value, sensory properties, texture, and shelf life are strongly influenced by its formulation. This includes factors such as the type of flour used, the addition of preservatives, and the choice of the leavening agent [4,5,6]. Understanding the production of bread and its nutritional profile has become increasingly important in the context of a rapidly growing global population [1,7]. As the demand for nutritious and sustainable food sources increases, this knowledge can contribute to developing improved formulations that can support global food security and meet the dietary needs of a larger, more diverse global population [1,7].

Bread is fundamentally produced from the flour of cereal grains (like wheat, rye, and oats) and/or the flour of pseudo-cereals and/or legumes, combined with water and a leavening agent. However, stored grains and pulses are prone to insect infestation, leading to annual losses estimated between 9% and 20% of total production [8].

*Sitophilus oryzae* L. (Coleoptera: Dryophtoridae) is a stored product pest of major economic importance, as it especially ravages wheat, barley, and maize but also oat, rye, millet, and buckwheat [9,10]. According to Singh and Sharma [11], *S. oryzae* attacks *Triticum aestivum* L. (soft wheat) more than *Triticum durum* Desf. (hard wheat) because the latter has lower protein, fiber, and total fat levels. Indeed, soft wheat varieties with high protein content are preferred by the beetle. Adults bore the kernel and feed on the carbohydrate-rich endosperm, while the tunneling larvae prefer the germ, of which they mainly consume proteins and vitamins [12].

Direct damage consists of a loss of nutritional and aesthetic value of the commodity, increased waste in the grain mass, and loss of technological properties of the obtained flour [13]. *S. oryzae* is also responsible for indirect damage by creating heat and moisture “pockets” in the stored grain mass and promoting premature germination and agglomeration into solid masses that make the product unusable [14]. Additionally, insect pest attacks can favor the development of mycotoxigenic fungi [15], with mycotoxin contamination considered one of the major concerns for food safety during grain storage [16].

Among the few synthetic insecticides approved for insect control in stored cereals, we can list pyrethroids (deltamethrin and cypermethrin), phosphine, and an organophosphate (pyrimiphos-methyl) [17]. Predictably, they all pose food and environmental safety issues and are leading to the emergence of resistance in insect populations [18,19,20].

In recent years, there have been increasing attempts to use safer and more eco-friendly strategies to overcome such side effects [21,22]. Diatomaceous earth (DE) is one of the active substances allowed in organic production protocols (Commission Implementing Regulation (EU) 2021/1165) [23]. DE is composed of unicellular eukaryotic fossil algae (Bacillariophyceae) having a silicon and aluminum, calcium, iron, and other mineral-oxide-rich external skeleton [24,25]. These fossils are abundant in aquatic and marine environments but also in terrestrial ecosystems such as mountains (e.g., Dolomites) where the sea once existed [26].

Still, research has also focused on botanically derived essential oils (EOs) as alternatives to synthetic pesticides. Both DE and EOs are effective against insects, but their use is restricted by respective limitations. In detail, DE, although legally admitted for the treatment of foodstuffs, is applied with difficulty in practical use, as it needs to be administered in high doses (up to 3.5 g/kg), and being abrasive, it damages the gears of machinery and may cause occupational allergies [27,28]. On the other hand, EOs have peculiar odors that can be transferred to the treated food, and having high volatility, their efficacy is short-lived [29].

Our attempt was to find a solution to the problem of over-dosing DE and make the EO smell acceptable for consumers when used to treat soft wheat against one of the most damaging beetles associated with stored food, namely *S. oryzae*.

To the best of our knowledge, there are only a few studies in the literature that apply DE in reduced doses (compared to the recommended label dose for preventive treatments), either alone or in combination with EO, against insect pests. Moreover, there is a lack of information regarding the impact of this treated wheat on the VOC profile and sensory acceptability of bakery products.

Therefore, this study aimed to test, under laboratory conditions, a possible new solution using a reduced dose of DE (1/8 or 1/16 of the recommended label dose), basil EO (*Ocimum basilicum* L.—Lamiaceae), or a combination of both at halved doses, to determine whether they could effectively control *S. oryzae* infestation in wheat. Additionally, the study investigated how these treatments would impact flour quality in bread making, in particular on the aroma and the organoleptic profile of the obtained bread. It also included a preliminary assessment of the effects of grain treatment on bread shelf life.

## 2. Materials and Methods

### 2.1. Materials

Food-grade *Triticum aestivum* L. suitable for bread making was purchased from the retailer Michelotti e Zei S.r.l. (Castelmartini, Potenza, Italy), 20 kg pack size, lot number: CL/2021/328/2, expiration date: June/2024.

The diatomaceous earth (DE) employed, commercialized as “SilicoSec”, was supplied by Biogard (CBC S.r.l., Grassobbio, Bergamo, Italy). It contains 50% particles smaller than 9.46 µm and is registered in Italy for insecticidal treatments on cereals.

Fresh samples of basil plants (*Ocimum basilicum* L.) were acquired from local markets in Ibarra, a city located in the Andean region of Ecuador. There, basil is cultivated at an average altitude of 2200 m above sea level, with moderate temperatures ranging from 18 °C to 25 °C and high light intensity due to its proximity to the equator. The basil leaves were air-dried at room temperature and subsequently subjected to EO extraction via steam distillation using a Clevenger apparatus for 3 h following ISO 11043:1998 and ISO 9235:2021 as previously reported [30,31]. The extracted EO was then dried over anhydrous sodium sulfate and stored in amber glass vials at 4 °C until use.

### 2.2. EO and HS-SPME Gas Chromatography–Mass Spectrometry Analysis

The EO was diluted to 5% in HPLC-grade n-hexane and then injected into a GC-MS apparatus. Gas chromatography–electron impact mass spectrometry (GC-EIMS) analyses were performed with an Agilent 7890B gas chromatograph (Agilent Technologies Inc., Santa Clara, CA, USA) equipped with an Agilent HP-5MS (Agilent Technologies Inc., Santa Clara, CA, USA) capillary column (30 m × 0.25 mm; coating thickness 0.25 μm) and an Agilent 5977B single quadrupole mass detector (Agilent Technologies Inc., Santa Clara, CA, USA). Analytical conditions were as follows: injector and transfer line temperatures of 220 and 240 °C, respectively; oven temperature programmed from 60 to 240 °C at 3 °C/min; carrier gas helium at 1 mL/min; injection of 1 μL (5% HPLC grade n-hexane solution); split ratio of 1:25. Acquisition parameters were as follows: full scan; scan range: 30–300 *m*/*z*; scan time: 1.0 s. The identification of the constituents was based on the comparison of their retention times with those of the authentic samples (when available), comparing their linear retention indices relative to the series of n-hydrocarbons (C6–C25). Computer matching was also used against a commercial [32] and a laboratory-developed mass spectra library built up from pure substances and components of commercial essential oils of known composition and MS literature data [33].

### 2.3. Sitophilus oryzae Rearing

*Sitophilus oryzae* specimens used for the toxicity tests were reared at the Entomology Laboratory of the Department of Agriculture, Food and Environment (DAFE) of the University of Pisa, as seen in Bedini et al. [34]. The species were maintained on soft wheat in polyethylene boxes (27 × 20 × 11 cm) closed with netted lids for ventilation. The substrate, used for nutrition and reproduction, was partially renewed every two weeks. The boxes were placed in a climatic chamber in the dark at 25 ± 1 °C and 65 ± 5% RH. To obtain homogeneous samples of insects in terms of age, adults were removed from the rearing box through gentle sifting with a sieve. As, after the emergence, the adults can remain up to 4 days in the kernels [9], the beetles found in the rearing boxes after 24 h were all, consequently, 0–4 days old. These specimens were used for the bioassays.

### 2.4. Toxicity Tests on Sitophilus oryzae

The experimental protocol, as well as the doses employed, were based on the work by Pierattini et al. [35]. The authors carried out a trial under operational conditions where, however, insect mortality could not be recorded due to the large mass of grain tested. Therefore, the toxicity tests in the current work were performed under laboratory conditions in polyethylene boxes (28 × 19 × 12.5 cm) closed with netted lids for ventilation. Each box contained 4 kg of food-grade soft wheat treated with:

- DE: 130 mg of DE/kg of wheat;
- EO: 130 µL of *O. basilicum* EO/kg of wheat;
- DE + EO: 65 mg of DE/kg of wheat + 65 µL of *O. basilicum* EO/kg of wheat;
- C: untreated control.

In fact, it was essential to work with sub-lethal doses in order to highlight the differences between the various treatments, as too high a mortality rate would have assimilated the treatments and confused the results. In detail, 130 mg of DE/kg of wheat represents roughly 1/8 of the recommended label dose for preventive treatments with DE (namely, 1 g/kg), and approximately the same dose was selected for the *O. basilicum* EO too (130 µL EO/kg of wheat). The treatment DE + EO contains half the dose of DE and EO compared to the two substances applied alone, so 65 mg of DE/kg of wheat (representing roughly 1/16 of the recommended label dose for preventive treatments with DE) and 65 µL EO/kg of wheat. DE and EO were manually mixed in a mortar just before use; all treatments were thoroughly distributed on soft wheat using a mechanical whisk.

Each test was repeated three times (*n* = 3) with 30 *S. oryzae* specimens of undetermined sex added to each box containing a specifically treated or control wheat sample. The 12 boxes were kept in a climatic chamber (in the dark at 25 ± 1 °C and 65 ± 5% RH) for one month. After this time, the content of each box was carefully sifted with a sieve to separate all the beetles from the wheat. Dead specimens were counted to calculate the mean mortality percentage, and live specimens were removed before the subsequent milling of the wheat.

### 2.5. Milling Procedure and Mycotoxins Test

The wheat grains were milled at the DAFE of the University of Pisa by a commercial mill (model Industry-Combi, Waldner Biotech, Lienz, Austria). Technical information about the mill is reported in Table S1. Each sample (in triplicate) of treated wheat (C, EO, DE, DE + EO) was milled separately to avoid contamination. Moreover, between one milling and the next, the mill was allowed to cool down at room temperature to avoid over-heating the flour. The mill is able to separate the different fractions of flour, and the type 0 flour (about 3 kg of flour for each treated wheat) was collected for bread making.

In view of the sensory analysis to be performed on the bread, after milling and before bread making, the presence of trichothecene mycotoxins was excluded. “DON and T-2 Mycotoxin Residues Rapid Test” kits (Bioeasy, Shenzhen, China) were used to detect the presence of deoxynivalenol (DON) and T-2 mycotoxins, according to the manufacturer’s instructions. Three replications were performed for each sample. First, 5 g of each flour sample was transferred into a 50 mL Falcon tube and added with 30 mL of 50% ethanol (*v*/*v*). The samples were mixed by vortexing for 30 s and then centrifuged at 4000 rpm for 2 min. The supernatant was collected and added to the diluent provided by the kits. For DON and T-2 tests, we followed the protocol for 400–500 ppb and 300–500 ppb detection limits, respectively. For all the flours tested, both kits (DON and T-2) gave negative results, and so we proceeded with the production of bread.

### 2.6. Bread Making

The bread-making procedure was performed with the “biga” method, a pre-ferment produced with baker’s yeast. The baker’s yeast was a commercially available compressed yeast (Zeus Iba S.r.l., Firenze, Italy). Baker’s yeast biga was obtained by mixing strong wheat flour type 0 (68% *w*/*w*), sterile water (31% *w*/*w*), and 1% (*w*/*w*) of baker’s yeast and subsequently left to ferment for 21 h at 18 °C as previously reported [36].

Four formulations of baker’s yeast bread, namely B-C, B-DE, B-EO, and B-DE + EO, were produced with water (32% *w*/*w*), leavening agent (biga) (15% *w*/*w*), salt (1% *w*/*w*), and flour (52% *w*/*w*). For each formulation, the flour used was obtained from the wheat treated as reported in Section 2.4.

The first leavening lasted 90 min at 26 ± 1 °C, then the dough was cut and shaped into 1 kg loaves which were left for 90 min at 32 ± 1 °C (second leavening). Finally, the loaves were baked at 220 °C for 60 min; after that, the bread was cooled at room temperature (23 ± 1 °C) and sliced (20 mm) for the sensory analysis.

### 2.7. Bread Shelf-Life Assessment

A preliminary assessment of the shelf life of bread was also carried out. For this purpose, four loaves (1 kg each), one for each of the four different flours being tested, were prepared; then 150 slices were packed separately in air (78.5% N_2_, 21% O_2_, 0.5% CO_2_). All the samples were checked daily for the presence of mold; each experimental run was stopped when 5% of the samples showed mold spoilage [1,37].

### 2.8. VOCs Profile of Bread

A solid phase micro-extraction (SPME) device (Supelco, St. Louis, MO, USA) coated with DVB/CAR/PDMS (100 μm) was used for sampling the headspaces. Both equilibration and sampling times were experimentally determined as 30 min, obtaining an optimal adsorption/absorption of volatiles and avoiding under- or over-saturation of the fiber and mass spectrometer detector. Once the sampling was finished, the fiber was withdrawn into the needle and transferred to the injection port of the GC-MS system. Blanks were performed before each first SPME extraction and randomly repeated during each series. Semi-quantitative comparisons of relative peak areas were performed between the same compounds in different samples. GC-MS conditions (analysis and identification) were identical to those used for EOs, except for splitless injection [36].

### 2.9. Sensory Analysis

The bread sensory profile was evaluated by a panel of 8 long-term members of the “Committee of Experts” of DAFE of the University of Pisa, according to the protocol previously described [38], including quantitative (i.e., percentage of white, presence of lacerations, dimension of alveoli and homogeneity of alveoli, olfactory intensity, frankness, wheat aroma, yeast aroma, acetic acid aroma, springiness, surface humidity, resistance to chewing, juiciness, cohesiveness, acidity, sapidity, bitterness, aftertaste, persistence, complexity of aroma, crunchiness, hardness, toasted aroma) and hedonic (i.e., visual attractiveness, olfactory pleasantness, tasting pleasantness, global pleasantness) indices. The overall hedonic index (HI) of bread was calculated according to Bianchi et al. [37]. The research received approval from the Ethics Committee of the University of Pisa (Comitato Bioetico dell’Università di Pisa, protocol n. 0088081/2023).

### 2.10. Statistical Analysis

Mortality data from the toxicity trials on *S. oryzae* were elaborated through one-way ANOVA using CoStat ver. 6.451 software (CoHort Software, Pacific Grove, CA, USA). Before that, normality (Shapiro–Wilk test *p* = 0.47) and homogeneity (Levene’s test *p* = 0.86) of data distribution were also checked. The mean values were separated by Tukey’s HSD post hoc test (*p* < 0.05).

The statistical analysis of volatile organic compounds was performed using one-way ANOVA (CoStat ver. 6.451, CoHort Software, Pacific Grove, CA, USA), and mean values were then separated by Tukey’s HSD post hoc test (*p* < 0.05). Furthermore, PCA and hierarchical cluster analysis (HCA) applying the Ward method and using two-way clustering were performed by the JMP Pro ver. 17.0 software (SAS Institute, Cary, NC, USA).

Sensory analysis data were processed by Big Sensory Soft 2.0 (ver. 2018, Centro Studi Assaggiatori, Brescia, Italy), carrying out a two-way ANOVA, with samples and panelist as main factors, followed by the Friedman test to individuate the main significant descriptors that allow significant discrimination of the samples [39].

## 3. Results and Discussion

### 3.1. Basil Essential Oil Composition

As reported in Table 1, the extracted basil EO exhibited a methyl chavicol chemotype: this phenylpropanoid dominated (75.2%) the composition of the EO, and it is commonly reported as a characteristic aroma contributor for this species [40], with its sweet, spicy, and anise-like odor [41]. Only two other compounds, among the identified seventeen, exhibited a relative abundance of over 1%: linalool accounted for 16.7%, while germacrene B for 2.9%. The former is a common constituent of basil EO, to which it confers a floral and sweet aroma [41].

Previous studies [42,43,44] have shown the repellent action of basil essential oil constituents against *Sitophilus oryzae* associated with common chemicals such as monoterpenoids, 1,8-cineole, α-pinene, carvone, and linalool, along with phenolic acids. Tapondjou et al. [43] confirmed that basil essential oils exhibit both toxic and repellent properties related to major components like methyl chavicol, linalool, ocimene, citronellol, geraniol, myrcene, pinene, and terpineol.

It is important to underline that the VOC composition of *Ocimum basilicum* essential oil was influenced by many factors, such as its origin [45,46,47]. However, four chemotypes of basil were identified [31,42,48,49,50] in relation to their main compounds regardless of their geographical origin. The basil EO used in the present research has been extracted from Ecuadorian plants and is a methyl-chavicol type, one of the most common chemotypes for this aromatic plant [31].

### 3.2. Mean Mortality Percentage of Sitophilus oryzae

**Figure 1:**
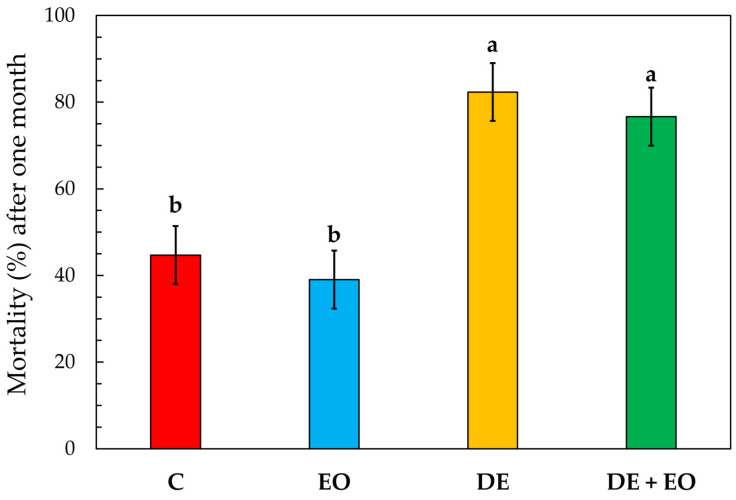
Mortality (%) of *Sitophilus oryzae* adults in the different treatments applied to soft wheat (C = control; EO = 130 µL of *Ocimum basilicum* essential oil/kg wheat; DE = 130 mg of diatomaceous earth/kg wheat; DE + EO = 65 mg of DE/kg wheat + 65 µL of *O. basilicum* EO/kg wheat) after one month. Bars represent standard errors (*n* = 3). Different letters indicate significant differences between treatments (Tukey’s HSD *p* < 0.05).

The mean mortality percentage caused by the three different treatments applied to soft wheat (and the untreated control) towards *S. oryzae* adults after one month is shown in Figure 1.

The one-way ANOVA analysis showed significant differences among the treatments (F3, 11 = 10.76, *p* = 0.003), as represented by the different letters in Figure 1 according to Tukey’s HSD post hoc test (*p* < 0.05). In detail, the most effective treatments were those involving DE: DE alone caused a mean mortality percentage ± standard error (*n* = 3) of 82.33 ± 6.70% and the combination of DE with *O. basilicum* EO (DE + EO) of 76.77 ± 6.70%. Although there was no statistically significant difference between the two treatments, it is of note that the dose of DE used was considerably different. Indeed, in the treatment with DE alone, the dose corresponded to roughly 1/8 of the recommended label dose for preventive treatments (namely, 1 g/kg), whereas, in the treatment with the mixture DE + EO, the DE dose used was roughly 1⁄16 of this dose. Conversely, the treatment with the EO alone, which resulted in a mean mortality percentage of 39.00 ± 6.70%, was not statistically different from the control (C), which showed a mean mortality percentage of 44.67 ± 6.70%.

Numerous studies confirm the effectiveness of DE to control stored product pests, with variable protection levels in correlation with temperature [51], earth particle size [52], cereal involved [53], and target species. Likewise, the efficacy of EOs, including some extracted from basil plants, has been thoroughly investigated over the years towards stored product pests [54,55,56,57]. In our trials, we obtained 38.67 ± 4.66% mortality of *S. oryzae* adults after one month of treatment with 130 µL *O. basilicum* EO/kg wheat; Popović et al. [55], using a similar dose of 150 µL basil EO/kg wheat, obtained 5.8% mortality but after only 48 h. For the congeneric species *Sitophilus granarius* L., Pierattini et al. [35] reported a median lethal dose of 434 µL of basil EO/kg wheat after nine days. However, this pest, although closely related to *S. oryzae*, might be less susceptible to basil EO.

Several attempts to exploit the synergy between the abrasive and desiccant properties of DE and the neurotoxic action of EOs have also been made. Similarly to our findings, *S. oryzae* and *Tribolium castaneum* Herbst (Coleoptera: Tenebrionidae) adults showed higher mortality when exposed to a mixture of garlic EO and DE [58] and *S. granarius* and *Tribolium confusum* Jacquelin du Val. when treated with a combination of ajowan EO and DE [59]. Conversely, Campolo et al. [60] found no advantages in mixing DE with sweet orange peel EO to manage *Rhyzopertha dominica* F. (Coleoptera: Bostrichidae). Lastly, although the insect pests’ mortality was not verified by Pierattini et al. [35] due to the impossibility of sieving 750 kg of wheat for each treatment, they assessed the presence of live insects. From their data, it is possible to find evidence of a higher and similar presence of beetles in the untreated control and the treatment with EO alone, whereas minimal presences (meaning higher mortality, as in our case) were detected when they applied DE alone and even lower ones with the mixture DE + EO.

Overall, our investigation further validated the abrasive and desiccant actions of DE that cause high mortality rates in insect pests. Moreover, it showed that it is possible to obtain a similar level of control when a reduced dose of DE is conveniently mixed with an EO having toxic properties on the targeted species. However, the EO alone, applied under these specific conditions and dose, was not sufficiently lethal, so a further reduction in the DE content in favor of a higher amount of EO would probably be useless.

### 3.3. Bread VOCs Profile

The control bread, both whole and sliced, was characterized by the usual volatiles of fresh wheat bread, i.e., alcohols, aldehydes/ketones, and esters (Table 2). They may derive from crust, crumb, or both [61]. They originate mostly from fermentation, Maillard reactions, and lipid oxidation [62].

In the whole bread samples, most of the volatiles came from the crust, mainly Maillard reactions, sugar caramelization, and thermal degradation products, i.e., pyrazines, pyridines, furans, etc. [36,62,63]. Indeed, in B-C-whole and B-DE-whole samples, nitrogen derivatives were higher than 20%, and carbonyl derivatives reached 46%. When these two samples were sliced, the crumb was exposed, and the above volatiles dropped to about 10 and 40%, respectively. However, the exposure of crumb allowed the release of products deriving from fermentation processes of dough sugars by yeasts and lactic acid bacteria, such as alcohols, aldehydes, and ketones [36,64]. For instance, alcohol levels increased from approximately 29 to 38% in these two samples. In particular, *Saccharomyces cerevisiae* fermentation produced high levels of isopentyl alcohol (from about 4% in whole bread to 10% in sliced) and 2-methyl butanol (from about 2% to 3%). Some other compounds appear to be produced in the crust and then transferred to the crumb [61], so they tended to increase in sliced samples, i.e., 2-pentyl furan (from 4.5 to 9%) and 3-methylbutanal (from 1 to 4%).

DE is an odorless, non-volatile silica powder and therefore cannot affect the VOCs in the mixture [65,66]. In fact, when comparing the results of bread with DE and control (Table 2) the addition of DE alone did not significantly affect the volatile bouquet. On the contrary, the results of the comparison between B-C and B-EO were different.

The addition of the EO to the grains strongly influenced the volatile emission of the bread samples. This was predictable due to the high volatility of the EO components. However, there is a lack of studies on the VOCs profile of bread made from cereals treated with EOs for pest control. Existing studies mainly refer to their use as a direct ingredient in bakery product formulations, for the production of active packaging materials, or for modifying the internal atmosphere of packaging to prolong bread shelf life [67,68,69,70].

In the present research, it seems that some of the oil components were able to resist the milling and baking processes and did not inhibit the microorganisms responsible for fermentation.

As a result, the volatile bouquet emitted by these samples (B-DE + EO and B-EO) was quite different, both for the whole and the sliced ones. In particular, phenylpropanoids become the main chemical class in the headspace, with methyl chavicol as the principal representative (about 36% to 44%). This result was predictable, as this compound was the major constituent of the EO (75.2%) [31]. The most volatile terpenes were principally detected in the sliced samples, probably because the crust prevents their loss, at least in part.

**Figure 2:**
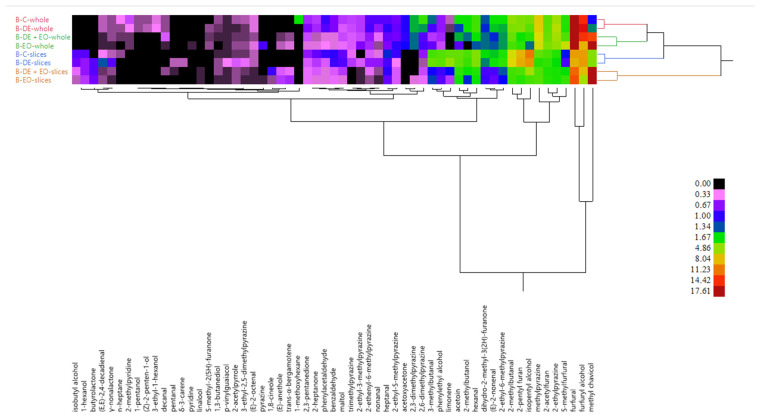
HCA of VOCs detected in the whole and sliced bread.

The HCA (Figure 2) and the PCA of VOCs (Figure S1) confirmed these observations, showing whole and sliced samples clustered separated from each other. Within each group, samples treated with the EO grouped separately from the non-treated ones.

### 3.4. Bread Sensory Profile

From the sensory analysis of the bread obtained using the four different flours investigated in this research, it emerged that the different treatments did not strongly influence the sensory profile of the product. This is also confirmed by visual analysis, where no significant differences were found for sensory analysis, as shown in the images of whole and sliced bread in Figure S2a,b.

**Figure 3:**
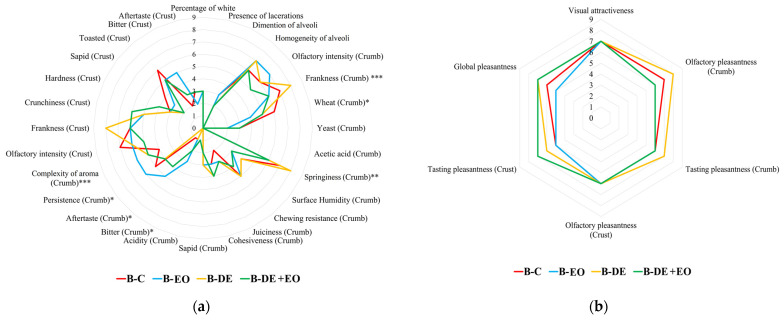
Sensory profile of bread: (**a**) Quantitative parameters; (**b**) Hedonic parameters. Significance level: *** = *p* < 0.001; ** = *p* < 0.01; * = *p* < 0.05; without asterisk = not significant (*p* ≥ 0.05).

As shown in Figure 3a, significant differences were observed only in a few parameters mainly related to the taste and retro-olfactory perception of the samples under examination.

In particular, both the use of DE and, especially, the addition of EO seem to reduce the perception of the wheat aroma in the crumb, which was more pronounced in the control sample. Conversely, in the crust, no significant differences were detected between the treatments and the control.

The use of EO gave the product a greater aromatic complexity together with a longer flavor persistence than the other samples. However, this difference was not significant when EO was combined with DE, compared to the control or bread treated solely with DE.

The use of a higher dose of EO (130 vs. 65 µL EO/kg of wheat) gave the product a more pronounced bitterness characteristic, probably contributing to increasing the negative perception of aftertaste. From a rheological perspective, bread produced with DE exhibited higher elasticity, while the addition of the EO appears to reduce the elastic properties of the crumb in both DE + EO and EO bread.

With respect to the hedonic results, as shown in Figure 3b, the DE treatment seemed to enhance the hedonic profile of the bread, particularly in terms of olfactory frankness of the crumb, which was significantly higher in this sample compared to the other treatments.

The combination of EO and DE tended to improve the taste pleasantness of the crust, while the smell pleasantness of the crust and the visual attractiveness remained consistent across the samples. Bread made with DE, whether used alone or in combination with EO, generally obtained higher evaluations in terms of overall pleasantness. Moreover, to assess the potential acceptability of the products obtained using DE and *O. basilicum* EO, the overall hedonic index (HI) of each bread was calculated. The minimum level selected for the acceptability is an HI value equal to 6.0 according to the previous studies [1,4,36,37,39].

**Figure 4:**
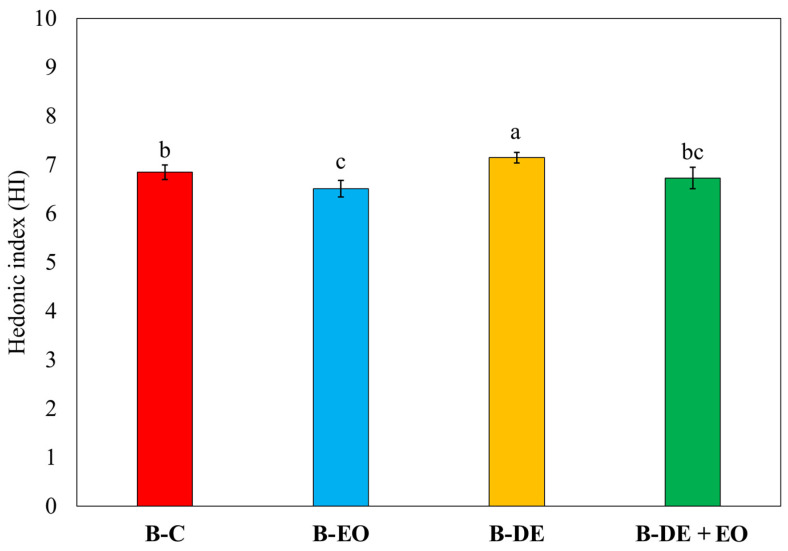
Hedonic index (HI). Bars represent standard errors (*n* = 8). Different letters indicate significant differences between treatments (Tukey’s HSD *p* < 0.05).

As shown in Figure 4, no statistically significant differences were observed with the presence or absence of EO in the bread.

The bread derived from DE-treated wheat achieved the highest level of acceptability, although the differences among samples were small, confirming that the treatments did not substantially alter the sensory profile of bread. This is consistent with previous results on the use of both EO and DE [67,71]. In general, the use of low doses of DE could help prevent changes in the physical and chemical characteristics of wheat that lead to a reduction in the technological quality of the flour [72]. Moreover, reduced doses of EO in bakery production could be both highly promising and potentially well-received by consumers. However, to the best of our knowledge, this specific topic has been explored in only a few studies. Most existing research focuses on the direct incorporation of EOs into bread formulations rather than their application for wheat protection [73,74].

### 3.5. Bread Shelf Life

The appearance of mold on the surface of bread slices was chosen as a parameter to define the limit of shelf life of bread during storage, and the test was considered to be concluded when more than 5% of the samples developed visible mold [1].

**Figure 5:**
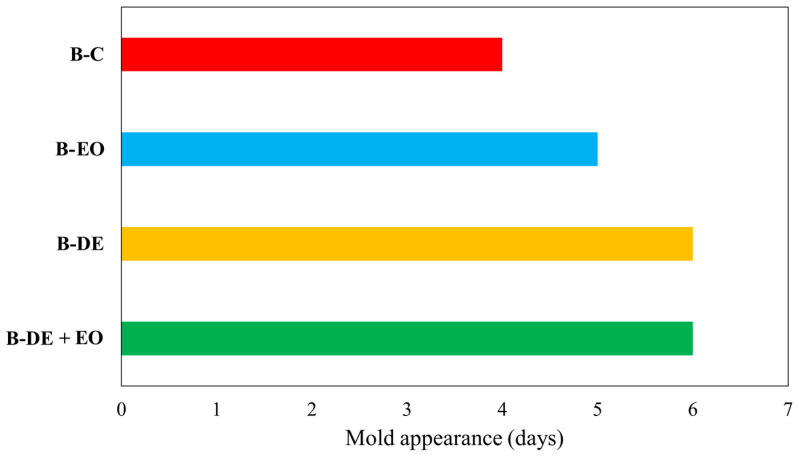
Mold appearance (days) in stored in air bread.

As shown in Figure 5, minor differences were found in the shelf life of the various samples compared to the control (B-C); however, treatments with DE and DE + EO extended the shelf life of the product by one day compared to the other sample. The anti-mold mechanism of essential oil in bread has been extensively studied [74,75,76]. Although there are no specific studies addressing the effect of DE on bread shelf life, some papers suggest that DE treatment may influence water absorption in flour [71]. This could have affected water availability for mold growth, however, further studies should be carried out to better understand the role of DE in bread shelf-life definition.

Furthermore, it is important to underline that this is sliced bread stored in air with short shelf life. As previously reported [1,4,36,37], the combination of these treatments with modified atmosphere packaging (MAP) could significantly extend bread shelf life.

## 4. Conclusions

To conclude, under the conditions adopted in the present study, the combined treatment of wheat with *O. basilicum* EO and DE at a significantly reduced dose (1⁄16 compared to the label dose for preventive treatments) provided good results in terms of *S. oryzae* mortality, statistically comparable to the use of DE alone in a double dose. This indicates a potential use of DE in combination with EOs for the protection of stored grain, allowing adherence to IPM programs and organic farming specifications. Furthermore, this protocol allows for a significant reduction in DE usage, minimizing its negative impact on machinery and, most importantly, on production chain operators.

Moreover, the treatment did not negatively affect the milling procedures or the yield of the process, as well as the technological properties of the flour and the sensory quality of the resulting bread. The sensory quality of bread made from flour derived from wheat treated with basil EO combined with DE was indeed well-received by the panel. The treatment increased the overall pleasantness of the bread, compared to the use of a double dose of EO alone, which released a strong anise aroma due to the presence of methyl chavicol, one of the major compounds of basil EO with a characteristic spicy, green, herbaceous aroma.

The shelf-life results have been encouraging, although these are very preliminary and further investigations are needed to fully capture changes in bread quality, beyond only visible mold spoilage, and to provide more comprehensive data.

Additionally, future trials will be designed for scaling up to verify the feasibility and the repeatability of the present research on a larger amount of treated wheat in silos. Moreover, consumer tests will be carried out to assess the acceptability of the final product.

## Figures and Tables

**Table 1:** Chemical composition of basil (*Ocimum basilicum*) essential oil.

Compounds	l.r.i. ^a^	Relative Abundance (%) ^b^
Limonene	1032	0.1 ± 0.07
1,8-Cineole	1034	0.4 ± 0.03
(*E*)-β-Ocimene	1052	0.1 ± 0.01
Linalool	1101	16.7 ± 0.03
Menthol	1173	0.3 ± 0.02
α-Terpineol	1189	0.2 ± 0.00
Methyl chavicol	1197	75.2 ± 0.59
Neral	1240	0.5 ± 0.05
*trans*-Citral	1273	0.8 ± 0.08
Eugenol	1358	0.1 ± 0.04
β-Caryophyllene	1420	0.6 ± 0.04
*trans*-α-Bergamotene	1438	0.9 ± 0.05
α-Humulene	1456	0.3 ± 0.02
(*E*)-β-Farnesene	1460	0.3 ± 0.04
Germacrene D	1478	0.5 ± 0.05
Germacrene B	1554	2.9 ± 0.06
*epi*-α-Cadinol	1641	0.1 ± 0.00
**Monoterpene hydrocarbons**		**0.2 ± 0.08**
**Oxygenated monoterpenes**		**18.8 ± 0.21**
**Sesquiterpene hydrocarbons**		**5.6 ± 0.26**
**Oxygenated sesquiterpenes**		**0.1 ± 0.00**
**Phenylpropanoids**		**75.3 ± 0.55**
**Total identified (%)**		**100.0 ± 0.00**

Values are presented as the average ± standard deviation (SD) of 3 samples. ^a^ Linear retention index on an HP5-MS capillary column; ^b^ Detection threshold ≥ 0.1%.

**Table 2:** VOCs detected (relative abundance (%)) in sliced and whole bread.

Volatile Organic Compounds (VOCs)	*p*-Value ^1^	B-C-Whole	B-DE-Whole	B-DE + EO-Whole	B-EO-Whole	*p*-Value ^1^	B-C-Slices	B-DE-Slices	DE + EO-Slices	EO-Slices
δ-3-Carene	ns	n.d.	n.d.	n.d.	n.d.	**	n.d. ^b^	0.3 ± 0.1 ^a^	n.d. ^b^	n.d. ^b^
Limonene	*	0.3 ± 0.1 ^a^	0.2 ± 0.1 ^a^	n.d. ^b^	n.d. ^b^	***	3.8 ± 0.1 ^b^	4.9 ± 0.1 ^a^	1.2 ± 0.1 ^c^	0.6 ± 0.1 ^d^
**Monoterpene hydrocarbons**	*	**0.3 ± 0.1 ^a^**	**0.2 ± 0.1 ^a^**	**n.d. ^b^**	**n.d. ^b^**	***	**3.8 ± 0.1 ^b^**	**5.1 ± 0.1 ^a^**	**1.2 ± 0.1 ^c^**	**0.6 ± 0.1 ^d^**
1,8-Cineole	ns	n.d.	n.d.	n.d.	n.d.	***	n.d. ^c^	n.d. ^c^	1.0 ± 0.0 ^a^	0.2 ± 0.1 ^b^
Linalool	ns	n.d.	n.d.	n.d.	n.d.	***	n.d. ^b^	n.d. ^b^	0.1 ± 0.0 ^a^	0.1 ± 0.0 ^a^
**Oxygenated monoterpenes**	**ns**	**n.d.**	**n.d.**	**n.d.**	**n.d.**	***	**n.d. ^c^**	**n.d. ^c^**	**1.1 ± 0.0 ^a^**	**0.3 ± 0.1 ^b^**
*trans*-α-Bergamotene	***	n.d. ^c^	n.d. ^c^	0.2 ± 0.0 ^b^	0.3 ± 0.0 ^a^	***	n.d. ^c^	n.d. ^c^	0.4 ± 0.0 ^b^	0.6 ± 0.0 ^a^
**Sesquiterpene hydrocarbons**	***	**n.d. ^c^**	**n.d. ^c^**	**0.2 ± 0.0 ^b^**	**0.3 ± 0.0 ^a^**	***	**n.d. ^c^**	**n.d. ^c^**	**0.4 ± 0.0 ^b^**	**0.6 ± 0.0 ^a^**
Pyrazine	***	n.d. ^b^	n.d. ^b^	n.d. ^b^	0.9 ± 0.0 ^a^	**	n.d. ^b^	n.d. ^b^	n.d. ^b^	0.1 ± 0.0 ^a^
Pyridine	*	n.d. ^b^	n.d. ^b^	n.d. ^b^	0.1 ± 0.0 ^a^	ns	n.d.	n.d.	n.d.	n.d.
2-Methylpyridine	*	0.5 ± 0.1 ^a^	0.4 ± 0.1 ^a^	0.2 ± 0.1 ^ab^	0.2 ± 0.0 ^b^	ns	n.d.	n.d.	n.d.	n.d.
Methylpyrazine	**	7.4 ± 0.3 ^a^	7.2 ± 0.0 ^a^	6.4 ± 0.1 ^b^	6.2 ± 0.2 ^b^	**	3.7 ± 0.0 ^a^	2.4 ± 0.1 ^b^	3.4 ± 0.4 a	3.4 ± 0.1 ^a^
2,6-Dimethylpyrazine	***	2.8 ± 0.1 ^a^	1.5 ± 0.1 ^b^	0.9 ± 0.2 ^c^	0.9 ± 0.0 ^c^	*	0.7 ± 0.1 ^a^	0.2 ± 0.0 ^c^	0.5 ± 0.1 ^ab^	0.4 ± 0.0 ^b^
2-Ethylpyrazine	**	5.1 ± 0.1 ^b^	5.6 ± 0.1 ^a^	5.2 ± 0.1 ^b^	4.8 ± 0.1 ^b^	*	2.3 ± 0.1 ^a^	1.7 ± 0.1 ^b^	2.1 ± 0.2 ^ab^	2.1 ± 0.1 ^ab^
2,3-Dimethylpyrazine	ns	1.6 ± 0.1	1.6 ± 0.1	1.4 ± 0.1	1.4 ± 0.1	***	n.d. ^b^	n.d. ^b^	n.d. ^b^	0.3 ± 0.0 ^a^
2-Ethyl-6-methylpyrazine	*	1.8 ± 0.1 ^a^	1.6 ± 0.1 ^ab^	1.5 ± 0.0 ^b^	1.5 ± 0.0 ^b^	***	2.2 ± 0.1 ^b^	2.6 ± 0.1 ^a^	1.4 ± 0.1 ^c^	1.2 ± 0.1 ^c^
2-Ethyl-5-methylpyrazine	*	0.8 ± 0.1 ^b^	0.8 ± 0.0 ^ab^	0.8 ± 0.1 ^b^	1.0 ± 0.0 ^a^	*	1.0 ± 0.2 ^a^	0.4 ± 0.1 ^b^	0.5 ± 0.1 ^b^	0.5 ± 0.1 ^b^
Trimethylpyrazine	ns	0.5 ± 0.0	0.5 ± 0.0	0.5 ± 0.1	0.5 ± 0.1	***	0.4 ± 0.0 ^b^	0.6 ± 0.1 ^a^	n.d. ^c^	n.d. ^c^
2-Ethyl-3-methylpyrazine	ns	0.6 ± 0.1	0.6 ± 0.1	0.5 ± 0.1	0.7 ± 0.0	***	n.d. ^c^	0.5 ± 0.1 ^a^	0.1 ± 0.0 ^b^	0.1 ± 0.0 ^b^
2-Ethenyl-6-methylpyrazine	**	0.9 ± 0.1 ^ab^	0.9 ± 0.0 ^a^	0.7 ± 0.1 ^bc^	0.6 ± 0.0 ^c^	**	0.5 ± 0.1 ^ab^	0.7 ± 0.1 ^a^	0.4 ± 0.0 ^b^	0.2 ± 0.0 ^c^
2-Acetylpyrrole	ns	0.2 ± 0.0	0.1 ± 0.0	0.2 ± 0.1	0.2 ± 0.0	*	0.2 ± 0.1 ^b^	0.5 ± 0.1 ^a^	0.2 ± 0.0 ^b^	0.2 ± 0.0 ^b^
3-Ethyl-2,5-dimethylpyrazine	ns	0.2 ± 0.1	0.2 ± 0.1	0.2 ± 0.1	0.2 ± 0.0	*	0.2 ± 0.1 ^b^	0.4 ± 0.0 ^a^	0.2 ± 0.1 ^b^	0.1 ± 0.0 ^b^
**Nitrogen derivatives**	***	**22.1 ± 0.2 ^a^**	**20.7 ± 0.0 ^b^**	**18.1 ± 0.5 ^d^**	**19.1 ± 0.2 ^c^**	**	**11.0 ± 0.1 ^a^**	**9.6 ± 0.0 ^b^**	**8.6 ± 0.5 ^c^**	**8.5 ± 0.4 ^c^**
Methyl chavicol	***	1.1 ± 0.1 ^d^	1.5 ± 0.1 ^c^	13.5 ± 0.3 ^b^	19.2 ± 0.7 ^a^	***	4.5 ± 0.4 ^c^	4.5 ± 0.1 ^c^	35.8 ± 1.0 ^b^	44.2 ± 0.5 ^a^
(*E*)-Anethole	*	n.d. ^b^	n.d. ^b^	0.2 ± 0.1 ^a^	0.3 ± 0.1 ^a^	***	n.d. ^b^	n.d. ^b^	0.5 ± 0.1 ^a^	0.6 ± 0.1 ^a^
**Phenylpropanoids**	***	**1.1 ± 0.1 ^d^**	**1.5 ± 0.1 ^c^**	**13.7 ± 0.2 ^b^**	**19.5 ± 0.8 ^a^**	***	**4.5 ± 0.4 ^c^**	**4.5 ± 0.1 ^c^**	**36.3 ± 1.1 ^b^**	**44.7 ± 0.4 ^a^**
*n*-Heptane	*	0.4 ± 0.1 ^a^	0.2 ± 0.0 ^ab^	0.2 ± 0.1 ^ab^	n.d. ^b^	***	0.2 ± 0.0 ^a^	n.d. ^b^	n.d. ^b^	n.d. ^b^
**Hydrocarbons**	*	**0.4 ± 0.1 ^a^**	**0.2 ± 0.0 ^ab^**	**0.2 ± 0.1 ^ab^**	**n.d. ^b^**	***	**0.2 ± 0.0 ^a^**	**n.d. ^b^**	**n.d. ^b^**	**n.d. ^b^**
3-Methylbutanal	**	1.4 ± 0.1 ^a^	0.9 ± 0.0 ^b^	0.6 ± 0.1 ^b^	0.8 ± 0.1 ^b^	***	3.1 ± 0.1 ^b^	3.9 ± 0.1 ^a^	1.1 ± 0.1 ^c^	1.3 ± 0.1 ^c^
2-Methylbutanal	**	4.8 ± 0.1 ^a^	4.4 ± 0.2 ^ab^	3.9 ± 0.1 b	3.2 ± 0.2 ^c^	***	6.2 ± 0.1 ^b^	6.8 ± 0.2 ^a^	3.1 ± 0.1 ^c^	2.9 ± 0.0 ^c^
2,3-Pentanedione	***	0.6 ± 0.0 ^b^	0.7 ± 0.0 ^a^	0.5 ± 0.0 ^c^	0.3 ± 0.0 ^d^	*	0.7 ± 0.1 ^a^	0.5 ± 0.1 ^ab^	0.4 ± 0.0 ^b^	0.4 ± 0.0 ^b^
Pentanal	ns	n.d.	n.d.	n.d.	n.d.	**	n.d. ^b^	0.3 ± 0.1 ^a^	0.1 ± 0.0 ^a^	n.d. ^b^
Acetoin	***	1.8 ± 0.1 ^a^	1.3 ± 0.0 ^b^	1.6 ± 0.1 ^a^	0.9 ± 0.1 ^c^	***	3.8 ± 0.2 ^a^	3.7 ± 0.1 ^a^	2.6 ± 0.1 ^b^	1.9 ± 0.1 ^c^
Hexanal	***	3.0 ± 0.2 ^a^	3.1 ± 0.1 ^a^	1.8 ± 0.1 ^b^	1.4 ± 0.2 ^b^	***	4.4 ± 0.1 ^a^	4.4 ± 0.1 ^a^	2.3 ± 0.1 ^b^	1.8 ± 0.1 ^c^
Dihydro-2-methyl-3(2*H*)-furanone	ns	1.4 ± 0.1	1.3 ± 0.1	1.4 ± 0.0	1.4 ± 0.1	***	1.7 ± 0.1 ^a^	1.5 ± 0.1 ^a^	0.9 ± 0.0 ^b^	1.0 ± 0.1 ^b^
Furfural	**	20.4 ± 0.1 ^b^	21.2 ± 0.2 ^b^	22.6 ± 0.4 ^a^	22.9 ± 0.4 ^a^	***	9.5 ± 0.1 ^c^	8.6 ± 0.1 ^d^	12.0 ± 0.3 ^b^	13.0 ± 0.1 ^a^
Acetoxyacetone	ns	1.1 ± 0.1	1.1 ± 0.1	1.0 ± 0.0	1.1 ± 0.0	ns	n.d.	n.d.	n.d.	n.d.
2-Heptanone	**	0.6 ± 0.1 ^a^	0.6 ± 0.0 ^a^	0.3 ± 0.1 ^b^	0.3 ± 0.0 ^b^	**	0.8 ± 0.0 ^a^	0.6 ± 0.1 ^b^	0.5 ± 0.1 ^bc^	0.3 ± 0.0 ^c^
Heptanal	**	0.9 ± 0.1 ^b^	1.0 ± 0.0 ^a^	0.8 ± 0.0 ^bc^	0.7 ± 0.0 ^c^	***	1.3 ± 0.1 ^a^	0.9 ± 0.0 ^bc^	1.0 ± 0.0 ^b^	0.8 ± 0.0 ^c^
2-Acetylfuran	ns	3.6 ± 0.1	3.7 ± 0.1	3.9 ± 0.4	4.3 ± 0.1	**	3.2 ± 0.1 ^a^	2.7 ± 0.1 ^b^	2.2 ± 0.1 ^c^	2.4 ± 0.2 ^bc^
5-Methyl-2(5*H*)-furanone	*	n.d. ^b^	0.2 ± 0.0 ^a^	0.2 ± 0.1 ^a^	0.2 ± 0.0 ^a^	ns	n.d.	n.d.	n.d.	n.d.
Benzaldehyde	***	0.7 ± 0.0 ^a^	0.7 ± 0.0 ^a^	0.3 ± 0.0 ^b^	0.3 ± 0.0 ^b^	***	1.0 ± 0.0 ^a^	1.0 ± 0.1 ^a^	0.5 ± 0.1 ^b^	0.4 ± 0.0 ^b^
5-Methylfurfural	***	2.3 ± 0.1 ^c^	2.3 ± 0.1 ^c^	3.3 ± 0.1 ^b^	5.9 ± 0.0 ^a^	***	1.2 ± 0.0 c	0.9 ± 0.1 ^d^	1.7 ± 0.2 ^b^	2.7 ± 0.1 ^a^
Phenylacetaldehyde	***	1.0 ± 0.1 ^a^	0.9 ± 0.1 ^a^	0.3 ± 0.0 ^b^	0.4 ± 0.1 ^b^	***	1.0 ± 0.1 ^a^	0.5 ± 0.1 ^b^	0.3 ± 0.0 ^c^	0.3 ± 0.0 ^c^
(*E*)-2-Octenal	ns	0.3 ± 0.0	0.3 ± 0.0	0.2 ± 0.0	0.3 ± 0.0	*	0.3 ± 0.1 ^a^	0.3 ± 0.1 ^a^	0.1 ± 0.0 ^b^	0.1 ± 0.0 ^b^
Nonanal	***	0.9 ± 0.1 ^a^	0.7 ± 0.1 ^a^	0.4 ± 0.0 ^b^	0.2 ± 0.0 ^b^	***	0.3 ± 0.0 ^b^	0.7 ± 0.1 ^a^	0.2 ± 0.0 ^bc^	0.1 ± 0.0 ^c^
(*E*)-2-Nonenal	*	1.8 ± 0.1 ^a^	1.6 ± 0.0 ^ab^	1.3 ± 0.1 ^b^	1.4 ± 0.1 ^b^	***	1.6 ± 0.0 ^b^	1.9 ± 0.1 ^a^	0.9 ± 0.0 ^c^	0.8 ± 0.0 ^c^
Decanal	**	0.2 ± 0.0 ^b^	0.2 ± 0.0 ^b^	0.5 ± 0.1 ^a^	0.1 ± 0.0 ^b^	**	n.d. ^b^	n.d. ^b^	0.2 ± 0.0 ^a^	n.d. ^b^
(*E*,*E*)-2,4-Decadienal	ns	0.3 ± 0.1	0.3 ± 0.1	0.1 ± 0.0	0.1 ± 0.0	***	0.4 ± 0.1 ^b^	1.4 ± 0.1 ^a^	0.2 ± 0.1 ^bc^	0.2 ± 0.0 ^c^
**Aldehydes/Ketones**	**ns**	**46.6 ± 0.1**	**46.0 ± 0.0**	**44.6 ± 0.8**	**45.9 ± 1.2**	***	**40.1 ± 0.1 ^a^**	**39.8 ± 0.2 ^a^**	**30.0 ± 0.4 ^b^**	**30.2 ± 0.1 ^b^**
Isobutyl alcohol	ns	n.d.	n.d.	n.d.	n.d.	**	0.9 ± 0.1 ^a^	0.8 ± 0.1 ^a^	0.4 ± 0.0 ^b^	0.3 ± 0.1 ^b^
Isopentyl alcohol	***	4.2 ± 0.1 ^a^	3.5 ± 0.1 ^b^	2.5 ± 0.1 ^c^	1.4 ± 0.1 ^d^	***	11.3 ± 0.1 ^a^	9.9 ± 0.1 ^b^	5.1 ± 0.1 ^c^	3.2 ± 0.1 ^d^
2-Methylbutanol	***	1.8 ± 0.1 ^a^	1.8 ± 0.0 ^a^	1.4 ± 0.0 ^b^	n.d. ^c^	***	3.2 ± 0.1 ^a^	2.9 ± 0.1 ^b^	1.7 ± 0.0 ^c^	1.3 ± 0.1 ^d^
1-Pentanol	***	0.2 ± 0.0 ^a^	0.2 ± 0.0 ^a^	n.d. ^b^	n.d. ^b^	ns	n.d.	n.d.	n.d.	n.d.
(*Z*)-2-Penten-1-ol	**	0.2 ± 0.0 ^a^	0.3 ± 0.1 ^a^	n.d. ^b^	n.d. ^b^	ns	n.d.	n.d.	n.d.	n.d.
1,3-Butanediol	*	0.2 ± 0.0 ^a^	0.2 ± 0.1 ^a^	0.1 ± 0.0 ^ab^	n.d. ^b^	ns	0.1 ± 0.0	0.2 ± 0.0	0.2 ± 0.0	0.1 ± 0.0
1-Methoxyhexane	***	1.7 ± 0.1 ^a^	n.d. ^b^	n.d. ^b^	n.d. ^b^	ns	n.d.	n.d.	n.d.	n.d.
Furfuryl alcohol	***	14.8 ± 0.2 ^b^	16.7 ± 0.1 ^a^	14.4 ± 0.1 ^b^	8.4 ± 0.0 ^c^	***	9.5 ± 0.1 ^a^	9.6 ± 0.1 ^a^	7.9 ± 0.3 ^b^	5.2 ± 0.0 ^c^
1-Hexanol	ns	n.d.	n.d.	n.d.	n.d.	*	0.9 ± 0.1 ^a^	0.7 ± 0.1 ^ab^	0.7 ± 0.1 ^ab^	0.6 ± 0.1 ^b^
2-Pentyl furan	***	4.4 ± 0.1 ^a^	4.5 ± 0.2 ^a^	1.8 ± 0.3 ^b^	2.5 ± 0.2 ^b^	***	8.4 ± 0.1 ^b^	8.8 ± 0.1 ^a^	3.3 ± 0.1 ^c^	2.4 ± 0.1 ^d^
3-Ethyl-1-hexanol	*	0.3 ± 0.1 ^a^	0.4 ± 0.1 ^a^	n.d. ^b^	n.d. ^b^	ns	n.d.	n.d.	n.d.	n.d.
Maltol	ns	0.6 ± 0.1	0.5 ± 0.1	0.5 ± 0.0	0.4 ± 0.0	***	0.8 ± 0.0 ^a^	0.9 ± 0.0 ^a^	0.6 ± 0.1 ^b^	0.3 ± 0.0 ^c^
Phenylethyl alcohol	ns	0.7 ± 0.1	0.7 ± 0.1	0.6 ± 0.0	0.5 ± 0.0	***	3.5 ± 0.5 ^a^	3.6 ± 0.1 ^a^	1.0 ± 0.1 ^b^	0.9 ± 0.1 ^b^
*p*-Vinylguaiacol	ns	0.1 ± 0.0	0.1 ± 0.0	0.1 ± 0.0	0.1 ± 0.0	**	0.1 ± 0.0 ^b^	0.4 ± 0.1 ^a^	0.1 ± 0.0 ^b^	0.1 ± 0.0 ^b^
**Alcohols/Phenols/Ethers**	***	**28.9 ± 0.1 ^a^**	**28.6 ± 0.4 ^a^**	**21.4 ± 0.1 ^b^**	**13.3 ± 0.1 ^c^**	***	**38.5 ± 0.5 ^a^**	**37.4 ± 0.2 ^b^**	**20.9 ± 0.3 ^c^**	**14.2 ± 0.2 ^d^**
Butyrolactone	ns	n.d.	n.d.	n.d.	n.d.	***	n.d. ^b^	0.9 ± 0.0 ^a^	0.9 ± 0.1 ^a^	0.8 ± 0.1 ^a^
γ-Nonalactone	ns	0.2 ± 0.0	0.2 ± 0.0	0.2 ± 0.0	0.1 ± 0.0	**	0.3 ± 0.1 ^b^	0.9 ± 0.1 ^a^	0.2 ± 0.1 ^bc^	0.1 ± 0.0 ^c^
**Lactones**	**ns**	**0.2 ± 0.0**	**0.2 ± 0.0**	**0.2 ± 0.0**	**0.1 ± 0.0**	***	**0.3 ± 0.1 ^c^**	**1.7 ± 0.1 ^a^**	**1.1 ± 0.1 ^b^**	**0.9 ± 0.1 ^b^**
**Total identified**		**99.5 ± 0.3**	**97.3 ± 0.2**	**98.2 ± 0.1**	**98.0 ± 0.1**		**98.2 ± 0.0**	**98.0 ± 0.2**	**99.3 ± 0.3**	**99.8 ± 0.1**

Values are presented as the average ± standard deviation (SD) of 3 samples. Different letters in a row for each group (whole and slices of bread) indicate statistical differences (Tukey’s HSD *p* < 0.05). ^1^ Significance level: *** = *p* < 0.001; ** = *p* < 0.01; * = *p* < 0.05; ns = not significant (*p* ≥ 0.05). n.d. = not detected.

## Data Availability

The original contributions presented in the study are included in the article/Supplementary Materials, further inquiries can be directed to the corresponding authors.

## Supplementary Materials

The following supporting information can be downloaded at: https://www.mdpi.com/article/10.3390/foods14040572/s1, Table S1: Technical information about the mill used to produce the flour; Figure S1: PCA of VOCs detected in the whole and sliced bread; Figure S2: Images of the bread produced: (a) whole bread; (b) sliced bread.
