# Supplementary material for: Reduced Doses of Diatomaceous Earth and Basil Essential Oil on Stored Grain Against the Wheat-Damaging Sitophilus oryzae: Influence on Bread Quality and Sensory Profile
Source: Foods. 2025 Feb 9;14(4):572. doi: 10.3390/foods14040572 (PMC11854187; doi:10.3390/foods14040572)
Supplement: Supplementary file 1 [file foods-14-00572-s001.zip › foods-3417181-supplementary.pdf]

Table S1. Technical information about the mill used to produce the flour.

Parameters	Desciption
Material	Wood-Stone Pine
Measurements (h/l/∅)	90/110/37 cm
Weight	60 kg
Mill output	240 g/min
Millstone diameter	150 mm
Millstone materials	Corundum/ceramic, self-sharpening
Hopper capacity	3 Kg
Grain mill motor	240 Volt/50 Hertz/1100 Watt
Sifting machine motor	240 Volt/50 Hertz/550 Watt

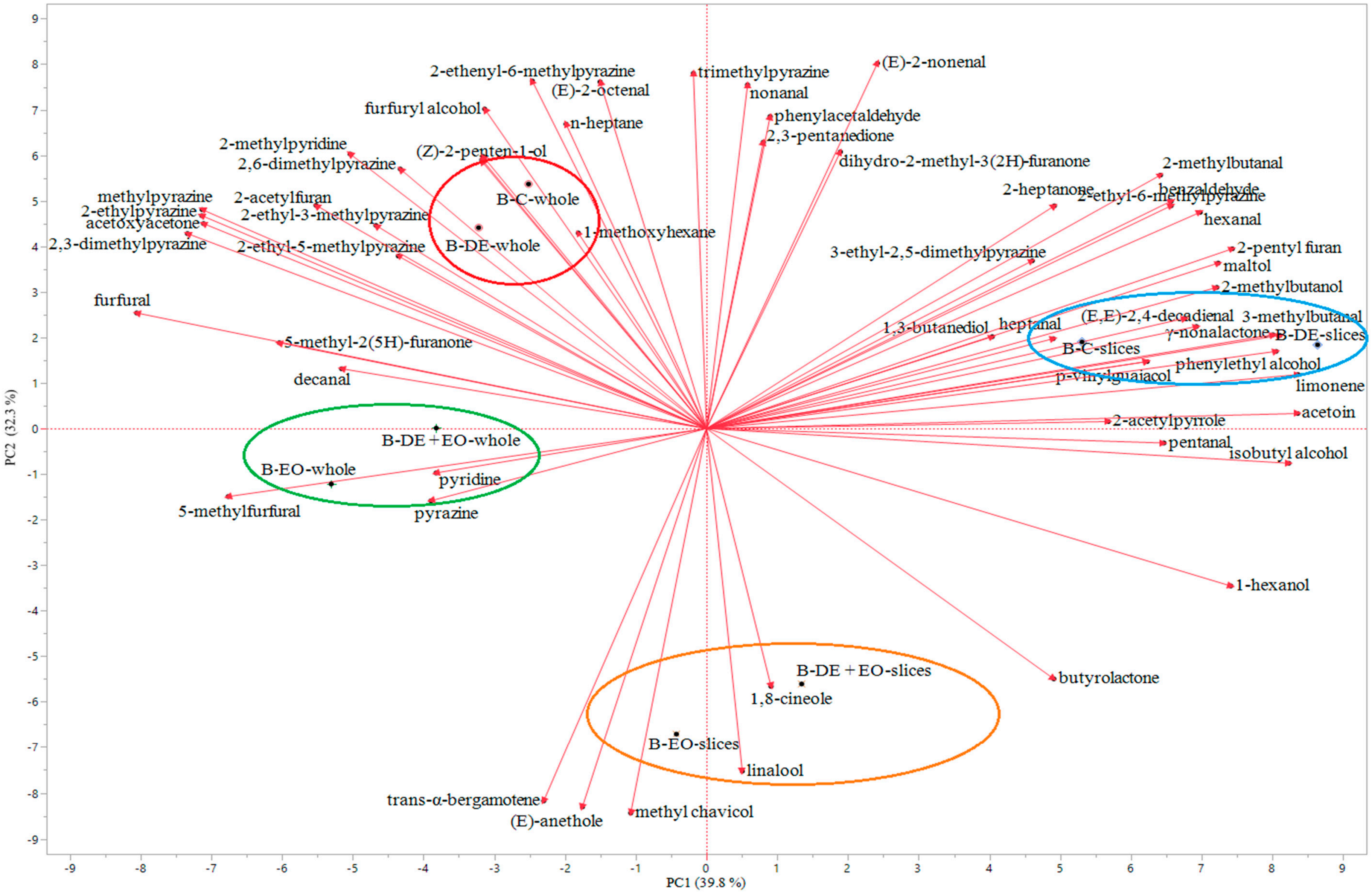

Figure S1. PCA of VOCs detected in the whole and sliced bread.

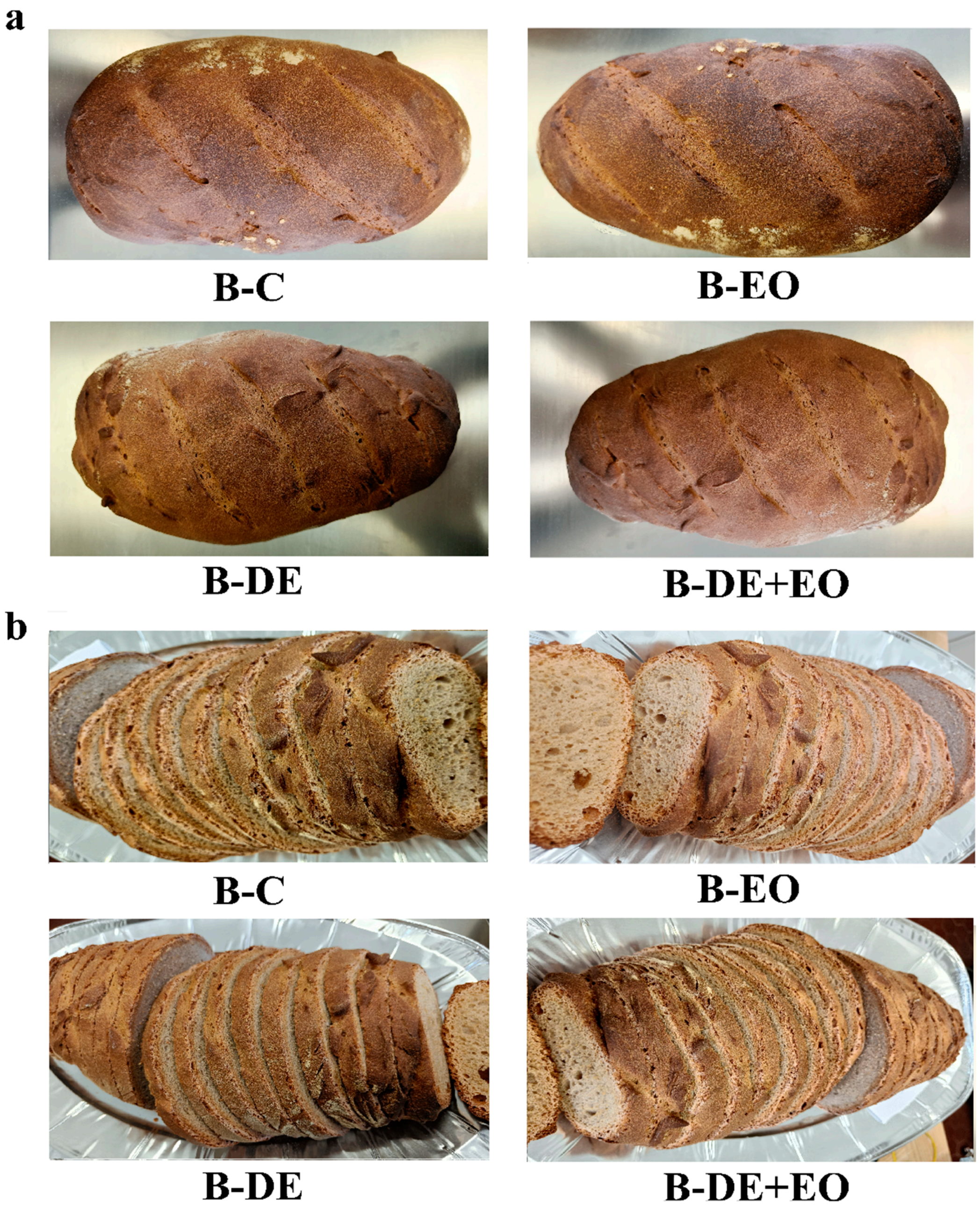

Figure S2. Images of the bread produced: (**a**) whole bread; (**b**) sliced bread.
